# Characterization data on the topical carrier DDC642

**DOI:** 10.1016/j.dib.2016.03.091

**Published:** 2016-04-01

**Authors:** Eline Desmet, Stefanie Bracke, Katrien Forier, Lien Taevernier, Marc C.A. Stuart, Bart De Spiegeleer, Koen Raemdonck, Mireille Van Gele, Jo Lambert

**Affiliations:** a Department of Dermatology, Ghent University, Ghent, Belgium; b Department of Pharmaceutics, Ghent University, Ghent, Belgium; c Center of Nano and Biophotonics, Ghent University, Ghent, Belgium; d Department of Pharmaceutical Analysis, Ghent University, Ghent, Belgium; e Electron microscopy, Groningen Biomolecular Sciences and Biotechnology Institute, University of Groningen, Groningen, The Netherlands

**Keywords:** Gene therapy, Lipid-based nanoparticle, Liposome, RNA interference, Topical drug delivery

## Abstract

This article contains original data, figures and methods used in the characterization of the liposomal carrier ‘DDC642’ for topical applications, described in “*An elastic liposomal formulation for RNAi-based topical treatment of skin disorders: proof-of-concept in the treatment of psoriasis*” (Desmet et al., 2016) [1]. Several elastic liposomal formulations have been evaluated for their ability to encapsulate and deliver RNA interference (RNAi) molecules to cultured primary skin cells. The efficiency and effectiveness of these liposomes were compared to that of our previously characterized liposomes, the ‘SECosomes’ (SEC) (Geusens et al., 2010) [2]. After selection of a potential superior carrier, based on encapsulation and transfection efficiency data (Desmet et al., 2016) [1], the selected DDC642 liposomes were characterized more in-depth. Herein, a detailed characterization of the DDC642 liposome and RNAi-loaded lipoplexes is given, including the matching protocols.

## Specifications table

**Table:** 

Subject area	*Biology*
More specific subject area	*Nanoparticles: elastic liposomes.*
Type of data	*Figure, table.*
How data were acquired	*Gel imaging system (ChemiDoc-It Imager, UVP or ChemiDoc XRS+, Bio-Rad), Cryo-electron microscopy (FEI Tecnai 20, FEI or CM120, Philips), Microplate reader (FLUOstar OPTIMA, BMG Labtech or EnVision Multilabel Plate Reader, Perkin Elmer), Flow cytometer (BD LSRFortessa, BD Biosciences), Real-time PCR detection system (MyiQ iCycler, Bio-Rad), Dynamic light scattering system (Zetasizer Nano series, Malvern).*
Data format	*Raw, analyzed.*
Experimental factors	*The RNAi encapsulation efficiency has been analyzed for several liposomal formulations. (Psoriasis-induced) keratinocytes and melanocytes were cultured in vitro prior to transfection with empty liposomes or loaded lipoplexes, to assess respectively cytotoxicity and transfection efficiency. Split thickness skin was prepared from thawed human skin, obtained from healthy volunteers, to use in permeation studies.*
Experimental features	*The encapsulation efficiency was evaluated using gel electrophoresis. in vitro transfection efficiency was analyzed by means of flow cytometry and Western blot. Franz diffusion cell permeation study was used to measure the skin penetration capacity. Cryo-TEM, CellTiter-Glo assay and dynamic light scattering was used to assess the morphology, cytotoxicity and particle size of the (loaded) liposomes, respectively.*
Data source location	*Ghent, Belgium.*
Data accessibility	*Data are included in this article.*

## Value of the data

• The data can be used as a guideline for other scientists investigating (topical) carrier systems for RNAi delivery.
• The data provide a more thorough characterization of the DDC642 liposomes, which hold great potential as topical delivery system.
• The data provided in this article could be helpful to researchers investigating gene therapy approaches in skin disorders.

## 1 Data

The DDC642 carrier was evaluated for its potential as topical RNAi delivery system by measuring the encapsulation efficiency, transfection efficiency and efficacy *in vitro*. Data is provided on the cytotoxicity of the empty liposomes and physicochemical and biological stability of the RNAi-loaded lipoplexes. Morphological data on the DDC642 liposomes and lipoplexes is provided as Cryo-TEM images.

## 2 Experimental design, materials and methods

### 2.1 Materials and reagents

Cy3^TM^ Dye-labeled anti-miR negative control #1 and Cy3^TM^ Dye-labeled pre-miR negative control #1 were purchased from Life Technologies (Ghent, Belgium). For additional product description, procedures regarding the liposome and complex formation, cell culture and *in vitro* transfection, we refer to the article of Desmet et al. [1].

### 2.2 Encapsulation efficiency by gel retardation assay

**Fig. 1:**
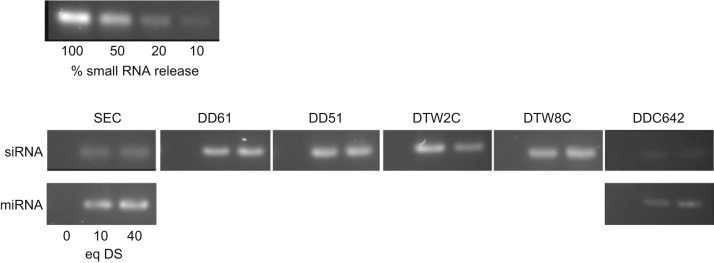
Images of the gel retardation assays using siRNA- and miRNA-containing complexes. For information about the composition and physicochemical parameters of the liposomes and corresponding lipoplexes see Table A in Ref. [1].

A 1.5% agarose gel containing 10% SYBR Safe (Life Technologies, Ghent, Belgium) was used to detect free, unbound RNAi molecules after complexation. After loading of the lipoplexes (LPX), electrophoresis was performed at 120 V during 45 min. Visualization was obtained by UV transillumination and gel photography using ChemiDoc XRS+ and Image Lab software (Bio-Rad, Eke, Belgium). Incubation of the LPXs with dextran sulfate (DS; Sigma-Aldrich, Bornem, Belgium) at anion charge excess (10 and 40 DS/RNAi (w/w)) for 30 min provided data on the amount of surface-bound RNA molecules and the complex stability (Fig. 1). Dilution series of ‘naked’ molecules were included to semi-quantify (Image J software) the amount of unbound or repelled molecules (*n*=3). Semi-quantitative data were presented in Ref. [1].

### 2.3 Morphological visualization

**Fig. 2:**
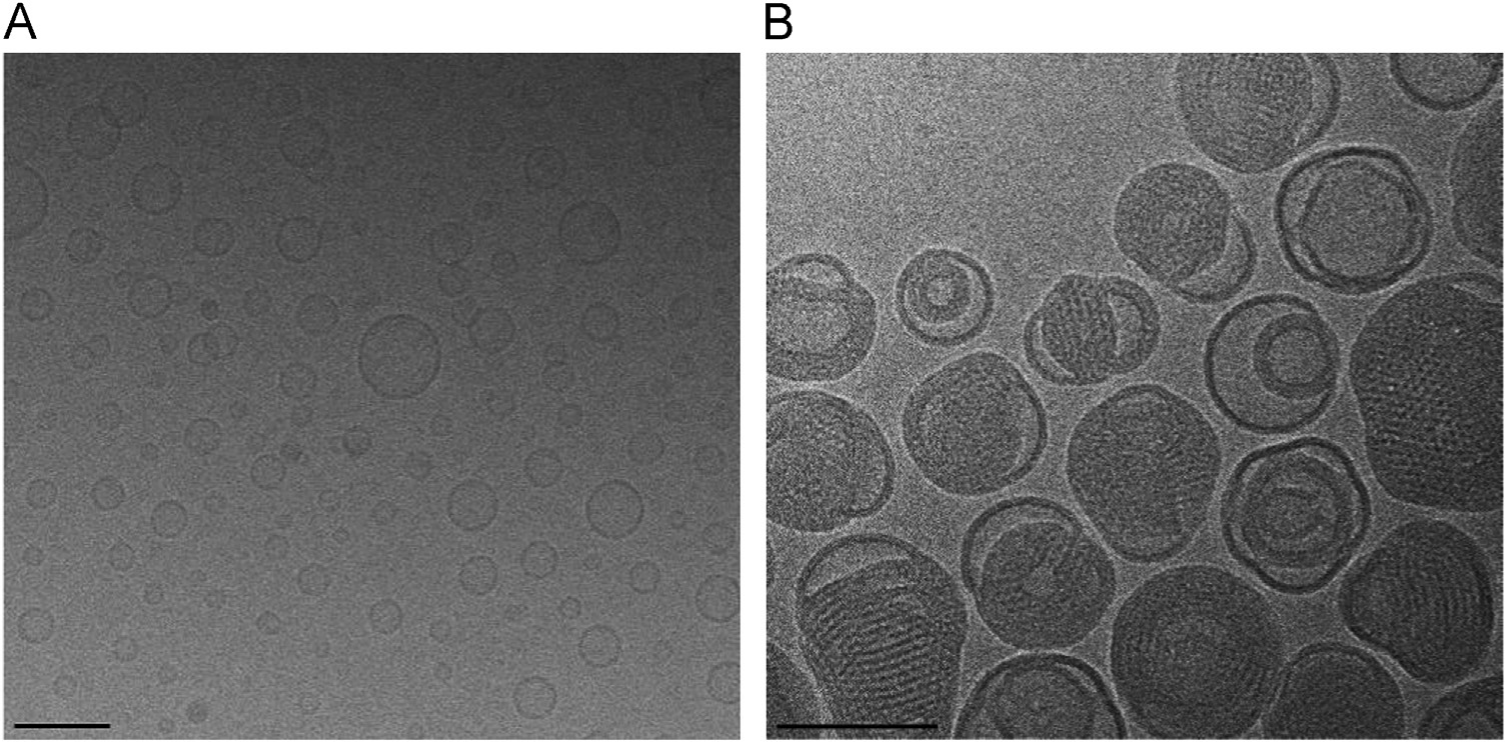
Cryo-TEM images of DDC642 liposomes (A) and lipoplexes (B). Scale bar:100 nm.

Cryogenic transmission electron microscopy (Cryo-TEM) was performed as described in Ref. [3]. Briefly, samples were placed on holey carbon-coated grids (Quantifoil 3.1/1), blotted and vitrified using a Vitrobot (FEI, Eindhoven, The Netherlands). Grids were observed in a FEI Tecnai 20 or a Philips CM120 electron microscope operating at 200 and 120 keV using a Gatan model 626 cryo-stage. Images were recorded on a slow-scan CCD camera under low-dose conditions (Fig. 2).

### 2.4 Post-transfection analysis of cells

Flow cytometry was used to determine the percentage of transfected cells after treatment (Table 1). Briefly, cells seeded in P60 dishes were treated with LPXs containing fluorescently labeled pre-miR, siRNA, or anti-miR, as described previously [1]. 48 h after LPX application, cells were washed with PBS and trypsinized. Following centrifugation (10 min, 1200 rpm), pellets were resuspended in PBS and placed on ice until flow analysis. Fluorescence was analyzed using a BD LSRFortessa (BD Biosciences, Erembodegem, Belgium). A minimum of 10,000 cells was analyzed in each measurement. FlowJo 9.7.6. software was used for analysis (Ashland, Oregon).

**Fig. 3:**
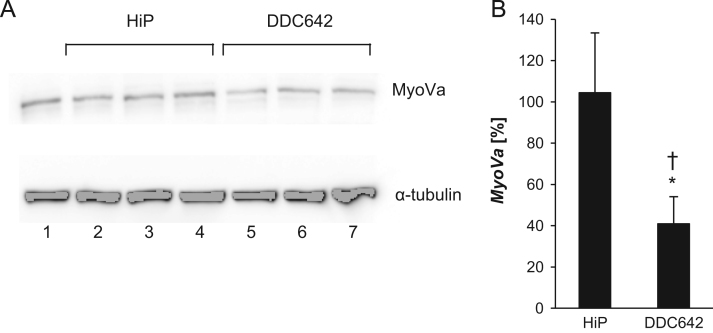
MyoVa protein expression after pre-miR-145 transfection of melanocytes. (A) Representative blot of untreated and treated cells. Lane 1: untreated; lane 2 and 5: empty vehicle; lane 3 and 6: pre-miR negative control #2; lane 4 and 7: pre-miR-145. (B) Quantitative analysis of the gels. Samples were compared to untreated control and α-tubulin was used as loading control. Error bars represent the mean±SD (*n*=3). *P*-values<0.05* compared to untreated control, † compared to HiPerfect.

The silencing efficacy of pre-miR-145-loaded liposomes in melanocytes was assessed by analyzing the protein expression of myosin Va (MyoVa), a direct target of miR-145 [4], by means of Western Blot. Forty-eight hours post-transfection, cultured melanocytes were lysed in RIPA buffer containing a protease inhibitor cocktail (Sigma-Aldrich, Diegem, Belgium). Protein concentration was determined using DC protein assay (Bio-Rad, Eke, Belgium) and Ultrospec 2100 pro UV/Visible spectrophotometer (GE Healthcare, Buckinghamshire, UK). Solubilization of total cellular extracts and immunoblotting was performed as described previously [5]. The primary antibody (anti-MyoVa exon F; Eurogentec, Liège, Belgium (1:10,000)) was incubated overnight at 4 °C. Scanned protein bands (ChemiDoc-It Imager, UVP, California, USA) were quantified with Image J software Fig. 3.

### 2.5 Cytotoxicity profile of the liposomes

**Fig. 4:**
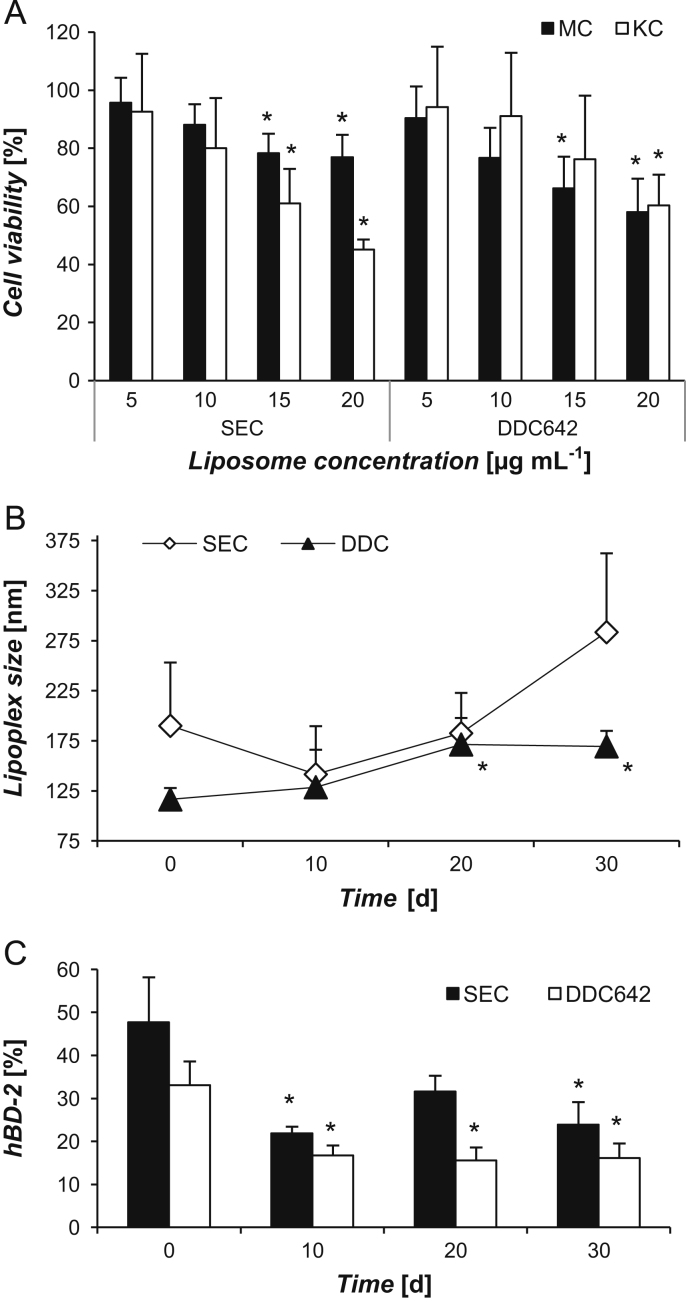
Cytotoxicity and stability assessment. (A) Cell viability of melanocytes (MC) and keratinocytes (KC) with increasing amounts of liposomes for 4 h. (B, C) Physicochemical and biological activity of lipoplexes during storage. The expression level of hBD-2 is relative to that of cells transfected with scrambled siRNA (100%). Data is shown as mean±SD (*n*≥3). *P*-values<0.05* compared to 5 µg mL^−1^ (A) or day 0 condition (B, C).

Cytotoxicity of the empty DDC642 liposomes was evaluated in cultured melanocytes (MC) and keratinocytes (KC) as described previously [2]. Quantification of the metabolic activity was carried out on a FLUOstar OPTIMA (BMG Labtech, Temse, Belgium). Cell viability was calculated as the percentage of luminescence compared to that of untreated cells (100%) (Fig. 4A).

### 2.6 Physicochemical and biological stability

Storage stability of the DDC642 lipoplexes was assessed as described earlier [2], and compared to that of the SECoplexes. Particle size was measured every 10th day during a period of 30 days using Zetasizer Nano series (Malvern, Worcestershire, UK) (Fig. 4B). In parallel, the biological stability was assessed *in vitro* in the psoriasis-induced keratinocyte model using liposomes containing siRNA directed against the *DEFB4* gene [1], [6]. The relative expression levels of hBD-2 in the transfected keratinocytes was obtained by RT-qPCR (performed as previously described [1]) (Fig. 4C).

### 2.7 *In vitro* Franz diffusion cell permeation study

The penetration of Cy5-labeled *DEFB4* siRNA lipoplexes through human skin was determined using a static Franz diffusion cell set-up with a 5 mL receptor compartment and an available diffusion area of 0.64 cm^2^ (Logan Instruments Corp., Somerset, New Jersey). Human skin from the abdominal and back region was collected from three healthy female patients (36±3 years old, mean±SEM) who had undergone cosmetic reduction surgery, with informed consent, approval from the ethical committee and confidentiality procedures in place (University Hospital, Ghent, Belgium). Skin preparation was done according to the internationally accepted guidelines [7] and as described previously [8]. Beside intact split-thickness skin samples (experimentally obtained thickness: 379±9 µm (mean±SEM)), damaged, tape-stripped skin (20 times with Scotch magic tape, 3M) was included to evaluate the use of a pre-treatment process but also for clinical purposes, *e.g.* an impaired skin barrier. Skin integrity analysis showed overall impedance values of 16.7±1.5 kΩ and 54.8±4.1 kΩ (mean±SEM) for respectively stripped and intact skin pieces, indicating significant skin damage by tape-stripping (*P*-value<0.05). Skin samples, epidermis facing upwards, were sandwiched between the donor and acceptor chambers, and the receptor fluid (0.01 M PBS, pH 7.4) was continuously stirred (600 rpm). Dose solutions (500 µL) from SEC and DDC642 lipoplexes were prepared with a final concentration of 1000 nM Cy5-siRNA and topically applied to the skin surface. The donor chamber was covered with parafilm and the temperature of the receptor compartment was kept at 32±1 °C. Samples (200 µL) were drawn at regular time intervals (up to 6 h) from the sampling port and replaced immediately. Samples were stored at −80 °C prior to analysis using the EnVision Multilabel Plate Reader (Perkin Elmer, Zaventem, Belgium). A determination limit of 50 pM was established. The experiments were replicated using 3 different skin donors, for both pre-treated skin (*n*=6) as well as intact skin (*n*=6). Data was presented in Ref. [1].

### 2.8 Statistical analysis

Statistical analysis of differences between 2 conditions was performed as described elsewhere, with a 0.05 level of probability (*P*-value<0.05) as level of significance [1].

## Figures and Tables

**Table 1:** Transfection efficiency of the liposomes in cultured primary skin cells. The percentages of transfected cells were analyzed 4 h post-treatment with a final concentration of 25 nm small RNA molecules (⁎) or 24 h with 50 nm (†). Grey colored: transfection conditions as described in Ref. [1] and Fig. 4C.

% transfected cells	siRNA^⁎^	*Pre-miR*^⁎^	*Anti-miR*^⁎^	*Anti-miR*^*†*^
*Psoriasis-induced KCs*				
HiPerfect	99.9	49.3	5.0	
SEC	99.9	41.7	40.8	
DDC642	99.9	46.8	12.0	
*Normal KCs*				
HiPerfect	99.9	20.7	37.2	29.2
SEC	99.9	16.6	63.0	94.2
DDC642	99.9	69.1	73.2	96.0
*Melanocytes*				
HiPerfect	99.9	30.9	8.14	
SEC	99.9	49.3	61.3	
DDC642	99.9	46.6	25.5	
