# Supplementary material for: Characterization data on the topical carrier DDC642
Source: Data Brief. 2016 Apr 1;7:1204–10. doi: 10.1016/j.dib.2016.03.091 (PMC4927961; doi:10.1016/j.dib.2016.03.091)
Supplement: Supplementary file 1 — Supplementary material [file mmc1.docx]

12/03/2016

Ghent, Belgium

Cover letter

Dear editor,

We confirm that the data in this manuscript has not been published previously and is not under consideration by another journal.

All authors have read and approved the manuscript and agree with submission to “Data in Brief”.

Moreover, the authors have no conflict of interest to declare. The authors state that there is no financial/personal interest or belief that could affect his/her objectivity. Detailed information regarding all financial and material support for the work is mentioned in the manuscript.

Kind regards,

Eline Desmet
